# Validation of a QTL for Grain Size and Weight Using an Introgression Line from a Cross between *Oryza sativa* and *Oryza minuta*

**DOI:** 10.1186/s12284-021-00472-1

**Published:** 2021-05-20

**Authors:** Yue Feng, Xiaoping Yuan, Yiping Wang, Yaolong Yang, Mengchen Zhang, Hanyong Yu, Qun Xu, Shan Wang, Xiaojun Niu, Xinghua Wei

**Affiliations:** Chinese National Center for Rice Improvement and State Key Laboratory of Rice Biology, China National Rice Research Institute, Hangzhou, 310006 China

**Keywords:** *Oryza sativa*, *Oryza minuta*, Introgression line, Grain size and weight, Quantitative trait loci

## Abstract

**Background:**

Grain size and weight are important target traits determining grain yield and quality in rice. Wild rice species possess substantial elite genes that can be served as an important resource for genetic improvement of rice. In this study, we identify and validate a novel QTL on chromosome 7 affecting the grain size and weight using introgression lines from cross of *Oryza sativa* and *Oryza minuta*.

**Results:**

An introgression line ‘IL188’ has been achieved from a wild species *Oryza minuta* (2n = 48, BBCC, W303) into *O. sativa japonica* Nipponbare. The F_2_ and F_2:3_ populations derived from a cross between IL188 and Nipponbare were used to map QTLs for five grain size traits, including grain length (GL), grain width (GW), grain length to width ratio (LWR), grain thickness (GT) and thousand grain weight (TGW). A total of 12 QTLs for the five grain traits were identified on chromosomes 1, 2, 3, 6, 7, and 8. The QTL-*qGL7* controlling GL on chromosome 7 was detected stably in the F_2_ and F_2:3_ populations, and explained 15.09–16.30% of the phenotypic variance. To validate the effect of *qGL7*, eight residual heterozygous line (RHL) populations were developed through selfing four F_2:3_ and four F_2:4_ plants with different heterozygous segments for the target region. By further developing SSR and Indel markers in the target interval, *qGL7* was delimited to a ~ 261 kb region between Indel marker Y7–12 and SSR marker Y7–38, which also showed significant effects on grain width and thousand grain weight. Comparing with the reference genome of Nipponbare, stop or frameshift mutations in the exon of the three putative genes *LOC_Os07g36830*, *LOC_Os07g36900* and *LOC_Os07g36910* encoding F-box domain-containing proteins may be the candidate genes for *qGL7*. Scanning electron microscopy analysis of the glume’s epidermal cells showed that the cell length and width of NIL-*qGL7*^IL188^ was higher than NIL-*qGL7*^Nip^, indicating that *qGL7* increases grain size and weight by regulating cell expansion.

**Conclusions:**

In this study, we detected 12 QTLs regulating grain size and weight using an introgression line from a cross between *Oryza sativa* and *Oryza minuta*. Of these loci, we confirmed and delimited the *qGL7* to a ~ 261 kb region. Three putative genes, *LOC_Os07g36830*, *LOC_Os07g36900* and *LOC_Os07g36910* encoding F-box domain-containing proteins may be the candidate genes for *qGL7*. These results provide a basis for map-based cloning of the *qGL7* gene and useful information for marker assisted selection in rice grain quality improvement.

**Supplementary Information:**

The online version contains supplementary material available at 10.1186/s12284-021-00472-1.

## Background

Rice (*Oryza sativa* L.) is one of the most important cereal crops in Asia and is the main staple food for the majority of peoples in the world. Breeding of high-yielding rice is crucial for meeting the food demand of the increasing world population (Ikeda et al. 2013). Grain yield in rice is determined by three major components: the number of panicles, the number of grains per panicle, and grain weight (Huang et al. 2013). Among these, the most reliable trait is grain weight, which is largely determined by grain size, which is specified by its three dimensions (length, width, and thickness) and the degree of filling (Xiong and Zhang 2010).

Grain size and weight are important components determining rice grain yield, and they are controlled by multiple quantitative trait loci (QTLs) (Zhang et al. 2012a; Yu et al. 2018). To date, over 400 QTLs modulating grain size and weight have been identified and are distributed on each of rice’s 12 chromosomes (Huang et al. 2013; Zuo and Li 2014; Kashif et al. 2020). However, only a few major QTLs including *GS3*, *qSW5*, *GW2*, *qGL3*/*GL3.1*, *GW8*, *GL7*/ *GW7*, *TGW6* and *GS9* have been isolated by map-based cloning methods (Mao et al. 2010; Shomura et al. 2008; Song et al. 2007; Qi et al. 2012; Zhang et al. 2012b; Wang et al. 2012; Wang et al. 2015a; Wang et al. 2015b; Ishimaru et al. 2013; Zhao et al. 2018). The isolation of these genes has enhanced our knowledge of the molecular regulatory mechanisms responsible for grain size and weight (Song and Ashikari 2008).

*Oryza minuta* (2n = 48, BBCC) is an allotetraploid wild species, which is endemic to Philippines and Papua New Guinea. This species belongs to the *Oryza officinalis* complex and harbors useful genes for resistance to blast blight, bacterial blight, brown planthopper and sheath blight (Amante-Bordeos et al. 1992; Brar and Khush 1997). However, low crossability and limited recombination between unrelated genomes limit the transfer QTLs from *Oryza minuta* to cultivars. Following the availability of advanced backcross quantitative trait loci (AB-QTL) approach proposed by Tanksley and Nelson (1996), several studies have been reported to identify the QTLs controlling yield and quality-related traits and to simultaneously transfer them from wild to cultivated species (Xiao et al. 1998; Thomson et al. 2003; Yoon et al. 2006; Tian et al. 2006; Mallikarjuna Swamy et al. 2012; Yun et al. 2016). However, few attempts have been made to identify and capture yield-related QTLs from *Oryza minuta* into cultivars.

**Fig. 1:**
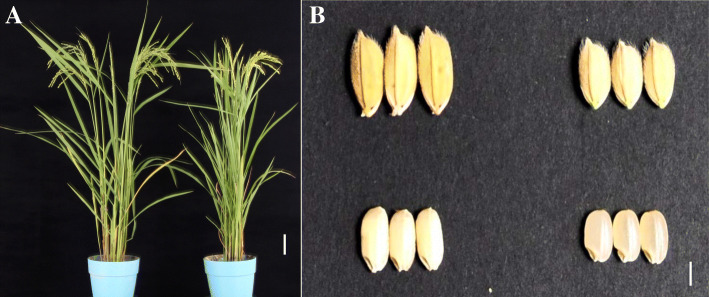
Comparison of whole-plant rice and grain performance between IL188 and Nipponbare. A, Plant type of IL188 (left) and Nipponbare (right). B, Paddy and brown rice grains of IL188 (left) and Nipponbare (right). Bars: 10 cm in A, 6 mm in B

In the present study, we used an advanced across line IL188 (Fig. 1) from a cross between the *japonica* variety, Nipponbare, *Oryza sativa* and a wild accession, W303, *Oryza minuta*, as the donor parent, to map QTLs for rice grain size traits. The objectives of this study were: (1) to reliably identify novel genomic regions associated with grain size traits from W303 (*Oryza minuta*), (2) to evaluate the effects of introgressive segments on grain size traits, (3) to fine mapping the QTL-*qGL7* and validate the effects of *qGL7* for rice grain size and weight on chromosome 7.

## Results

### Genetic Background of IL188

**Fig. 2:**
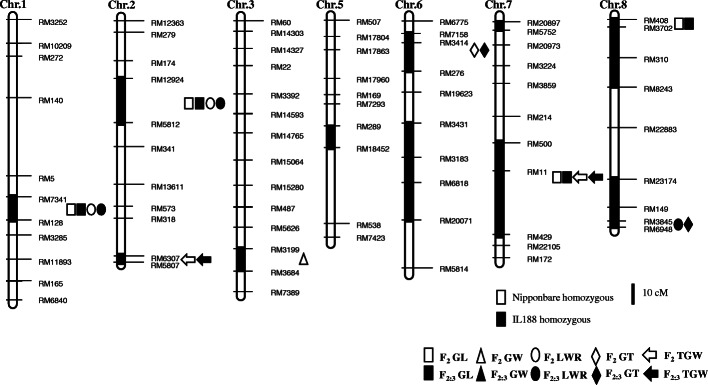
Genetic linkage map showing QTL positions detected in the F_2_ and F_2:3_ populations. White and black shapes indicate F_2_ and F_2:3_ population, respectively

A total of 512 SSR markers were screened for polymorphism between W303 and Nipponbare. Among them, 185 markers produced polymorphic bands between the parents. These 185 polymorphic markers were further used to assay the genotype of IL188. Thirty of these markers (16.2%) showed W303 genotype, which covered 11 regions distributing on seven chromosomes. The introgressed segments distributed on chromosomes 1, 2 (two), 3, 5, 6 (two), 7 (two), and 8 (two), respectively (Fig. 2). These 30 markers were further used to genotype the F_2_ and F_2:3_ populations derived from a cross between Nipponbare and IL188.

### Trait Performance of the Parents, F_2_ and F_2:3_ Populations

**Fig. 3:**
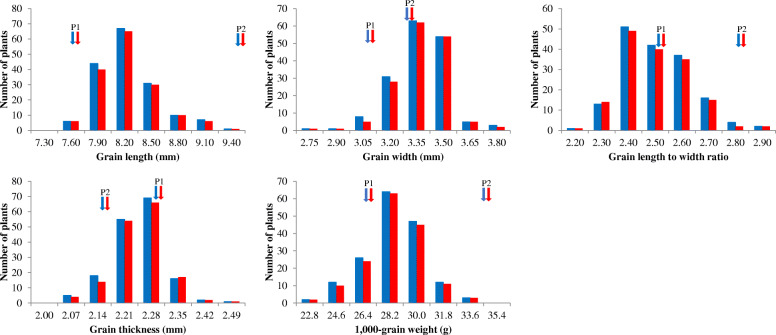
Frequency distribution of five grain shape traits in the F_2_ and F_2:3_ populations. (P_1_: Nipponbare, P_2_: IL188). The vertical axis of each figure represents the number of F_2_ and F_2:3_ plants, blue bars and red bars indicate F_2_ and F_2:3_, respectively

The phenotypic values of the two parents of five agronomic traits, including GL, GW, GT, LWR, and TGW were shown in Table 1. Compared with Nipponbare, IL188 had higher values for GL, GW, LWR, and TGW but lower values for GT. The frequency distributions of the five grain size traits in the F_2_ and F_2:3_ populations displayed a continuous variation (Fig. 3). All these traits expect GL and TGW showed two-way transgressive segregation and followed a near normal distribution in the both populations. The results fulfill the requirement of QTL mapping.

**Table 1:** The phenotypic performance of five grain size traits in IL188 and Nipponbare

Trait	Nip	IL188
GL (mm)	7.798 ± 0.060	9.416 ± 0.133^**^
GW (mm)	3.087 ± 0.040	3.347 ± 0.072^**^
LWR	2.526 ± 0.021	2.819 ± 0.042^**^
GT (mm)	2.295 ± 0.035	2.171 ± 0.020^**^
TGW (g)	24.10 ± 0.15	34.40 ± 0.19^**^

*Nip* Nipponbare, *GL* grain length, *GW* grain width, *LWR* the ratio of grain length to grain width, *GT* grain thickness, *TGW* thousand grain weight. ^**^ indicate significance at *P* < 0.01

### Correlation Analysis of Five Grain Traits

The correlation coefficients among the five grain traits in the F_2_ and F_2:3_ populations were shown in Table 2. The correlation coefficients ranged from − 0.677 to 0.614 and − 0.662 to 0.598, respectively, in the F_2_ and F_2:3_ populations. Significant correlation was observed for each pair-wise combination except that between GL and GT, LWR and TGW. In the both populations, GL showed positive correlation with GW, LWR and TGW, GW displayed strong positive correlation with GT and TGW, while LWR showed negative correlation with GW and GT.

**Table 2:** Coefficients of pairwise correlation among five grain size traits in the F_2_ and F_2:3_ populations

	GL	GW	LWR	GT	TGW
GL		0.276**	0.536**	− 0.025	0.598**
GW	0.309**		− 0.662**	0.470**	0.509**
LWR	0.488**	−0.677**		−0.439**	0.021
GT	0.025	0.515**	−0.460**		0.438**
TGW	0.614**	0.547**	−0.026	0.478**	

*GL* grain length (mm), *GW* grain width (mm), *LWR* the ratio of grain length to grain width, *GT* grain thickness (mm), *TGW* thousand grain weight (g). The data of the left lower and the top right triangles in the Table 2 are the correlation coefficients in F_2_ and F_2:3_ populations, respectively. ^**^ indicate significance at *P* < 0.01

### QTLs for Grain Size Traits in the F_2_ and F_2:3_ Populations

A total of 12 QTLs for five grain size traits were detected on chromosomes 1, 2, 3, 6, 7, and 8 in the F_2_ and F_2:3_ populations (Table 3, Fig. 2). The phenotypic variance explained by each QTL ranged from 4.72% to 16.30%. Four of these regions were found to affect two traits. The RM7341–RM128 interval on the chromosome 1 and RM12924–RM5812 interval on chromosome 2 showed consistent effects on GL and LWR in both populations. In the RM7341–RM128 interval, *qGL1* and *qLWR1* explained phenotypic variances by 8.77% and 7.55% in the F_2_ population, and 9.13% and 7.40% in the F_2:3_ population, respectively. In the RM12924–RM5812 interval, *qGL2* and *qLWR2* explained phenotypic variances by 6.90% and 5.02% in the F_2_ population, and 7.09% and 5.68% in the F_2:3_ population, respectively. The enhancing alleles of these QTLs all derived from IL188. The RM500–RM429 interval on chromosome 7 showed consistent effects on GL and TGW in the both populations. The *qGL7* and *qTGW7* explained phenotypic variances by 16.30% and 9.97% in the F_2_ population, and 15.09% and 6.65% in the F_2:3_ population, respectively. The enhancing alleles of the two QTLs also derived from IL188. The RM3845–RM6948 on chromosome 8 exhibited significant effects on GT and LWR only in the F_2:3_ population. The *qGT8* and *qLWR8* explained 7.41% and 4.72% of phenotypic variances, with enhancing alleles derived from Nipponbare and IL188, respectively. The other four regions, which covered RM6307–RM5807, RM3199–RM3684, RM7158–RM276, RM408–RM3702 on chromosome 2, 3, 6 and 8, respectively, were each detected for a single trait, with *R*^*2*^ ranging from 4.85% to 7.06%.

**Table 3:** QTLs detected for five grain size traits in F_2_ and F_2:3_ populations

Trait	QTL	Chr.	Interval	F_2_			F_2:3_		
				*LOD*	*A*	*R*^2^ (%)	*LOD*	*A*	*R*^2^ (%)
GL	*qGL1*	1	RM7341–RM128	4.55	−0.142	8.77	4.35	−0.144	9.13
GL	*qGL2*	2	RM12924–RM5812	4.20	−0.120	6.90	3.81	−0.123	7.09
GL	*qGL7*	7	RM500–RM429	7.34	−0.190	16.30	6.15	−0.180	15.09
GL	*qGL8*	8	RM408–RM3702	2.82	−0.066	5.13	2.65	−0.060	4.85
GW	*qGW3*	3	RM3199–RM3684	2.55	−0.047	5.77			
LWR	*qLWR1*	1	RM7341–RM128	2.92	−0.051	7.55	2.81	−0.050	7.40
LWR	*qLWR2*	2	RM12924–RM5812	2.69	−0.040	5.02	2.74	−0.043	5.68
LWR	*qLWR8*	8	RM3845–RM6948				2.52	−0.038	4.72
GT	*qGT6*	6	RM7158–RM276	4.26	0.021	5.68	4.96	0.032	6.25
GT	*qGT8*	8	RM3845–RM6948				2.59	0.024	7.41
TGW	*qTGW2*	2	RM6307–RM5807	2.57	−0.072	7.06	2.54	−0.070	6.75
TGW	*qTGW7*	7	RM500–RM429	4.56	−0.070	9.97	3.74	−0.061	6.65

*GL* grain length (mm), *GW* grain width (mm), *LWR* the ratio of grain length to grain width, *GT* grain thickness (mm), *TGW* thousand grain weight (g). *A*, Additive effect of *QTL* Positive value and negative value of additive effects represented the Nipponbare and IL188 alleles, respectively. *R*, variance explained by the QTL

Among these regions, the RM500–RM429 interval on chromosome 7 showed the largest effect for GL and relatively stable QTLs for TGW. Therefore, the region was chosen for further validation. For ease of description, the *qGL7* and *qTGW7* detected in this region were integrated as *qGL7*.

### Substitution Mapping of *qGL7* and Sequence Analysis of Candidate Genes

**Fig. 4:**
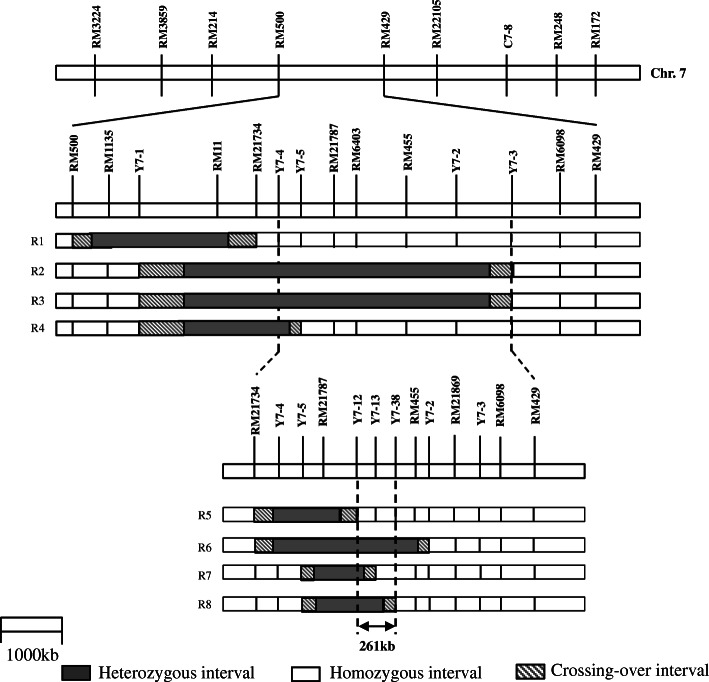
Genotypes compositions of NIL populations in the target region. NIL, near-isogenic line

Four NIL-F_2_ populations carrying heterozygous segments overlapped in the RM500–RM429 interval were constructed, including R1, R2, R3 and R4. Significant genotypic effects were detected for the three grain size traits in R2 and R3. In the two populations, the additive effects were 0.115 and 0.109 for GL, 0.065 and 0.050 for GW, 0.621 and 0.907 for TGW, explained phenotypic variances by 19.54% and 15.46%, 21.65% and 10.24%, and 16.09% and 16.86% (Table 4). The enhancing allele was derived from IL188, the same as what was found in the F_2_ and F_2:3_ populations. The additive effects and *R*^*2*^ were similar between R2 and R3, indicated that *qGL7* located in the common segregating regions of the two populations. In R1 and R4, no significant effect was detected for any trait, indicated that *qGL7* located outside of segregating regions of the two populations. As shown in Fig. 4, this is an interval flanked by markers Y7–3 and Y7–4, corresponding to a 725-kb region in the Nipponbare genome.

**Table 4:** QTLs detected for three grain traits in the R1–R8 populations

Population	Marker interval	Trait	*LOD*	*A*	*R*^*2*^*(%)*
R1	RM1135–RM11	GL	0.27	− 0.038	1.41
		GW	1.24	0.041	4.61
		TGW	0.38	0.039	1.38
R2	RM11–Y7–2	GL	7.35	−0.115	19.54
		GW	9.37	−0.065	21.65
		TGW	11.00	−0.621	16.09
R3	RM11–Y7–2	GL	5.07	−0.109	15.46
		GW	3.29	−0.050	10.24
		TGW	7.16	−0.907	16.86
R4	RM11–RM21734	GL	0.32	−0.041	1.80
		GW	1.03	0.002	0.06
		TGW	0.26	0.141	1.03
R5	Y7–4–RM21787	GL	0.45	−0.025	1.39
		GW	0.14	0.001	0.04
		TGW	0.30	−0.096	0.48
R6	RM21787–Y7–13	GL	17.99	−0.129	48.52
		GW	4.08	−0.026	16.44
		TGW	8.04	−0.595	25.38
R7	RM21787–Y7–12	GL	1.91	−0.077	7.33
		GW	1.25	−0.007	0.40
		TGW	1.72	−0.263	1.87
R8	Y7–4–RM455	GL	5.81	−0.074	18.12
		GW	3.61	−0.031	12.77
		TGW	3.21	−0.494	9.22

*GL* grain length (mm), *GW* grain width (mm), *TGW* thousand grain weight (g). *A*, additive effect, Positive value and negative value of additive effects represented the Nipponbare and IL188 alleles, respectively. *R*, variance explained by the QTL

Following the update target regions, other four NIL-F_2_ populations were developed, including R5, R6, R7 and R8. Significant genotypic effects were detected in R6 and R8, but not in R5 and R7. In R6 and R8, the additive effects were 0.129 and 0.074 for GL, 0.026 and 0.031 for GW, 0.595 and 0.494 for TGW, explained phenotypic variances by 48.52% and 18.12%, 16.44% and 12.77%, and 25.38% and 9.22% (Table 4). Again, the enhancing allele was derived from IL188. These results indicated that *qGL7* was located within the common segregating regions of R6 and R8 but outside the segregating regions of R5 and R7. Consequently, *qGL7* was delimited into a 261-kb region flanked by Y7–12 and Y7–38 (Fig. 4).

In the 261-kb genomic region of the Nipponbare genome, a total of thirty-seven putative genes were predicted based on the Nipponbare sequence (Os-Nipponbare-Reference-IRGSP-1.0). Whole-genome resequencing was performed on the NIL-*qGL7*^IL188^. Compared with the Nipponbare reference genome, seven stop code mutations and ten frameshift mutations were identified in the exonic region of the twelve genes (Table S3). One of them, *LOC_Os07g36850* encodes a putative transposon protein, four genes encode retrotransposon proteins and four genes encode expressed protein of unknown function. In addition, *LOC_Os07g36900* encodes a protein containing F-box and LRR motifs, both *LOC_Os07g36830* and *LOC_Os07g36910* encode F-box proteins.

### Histocytological Analysis

**Fig. 5:**
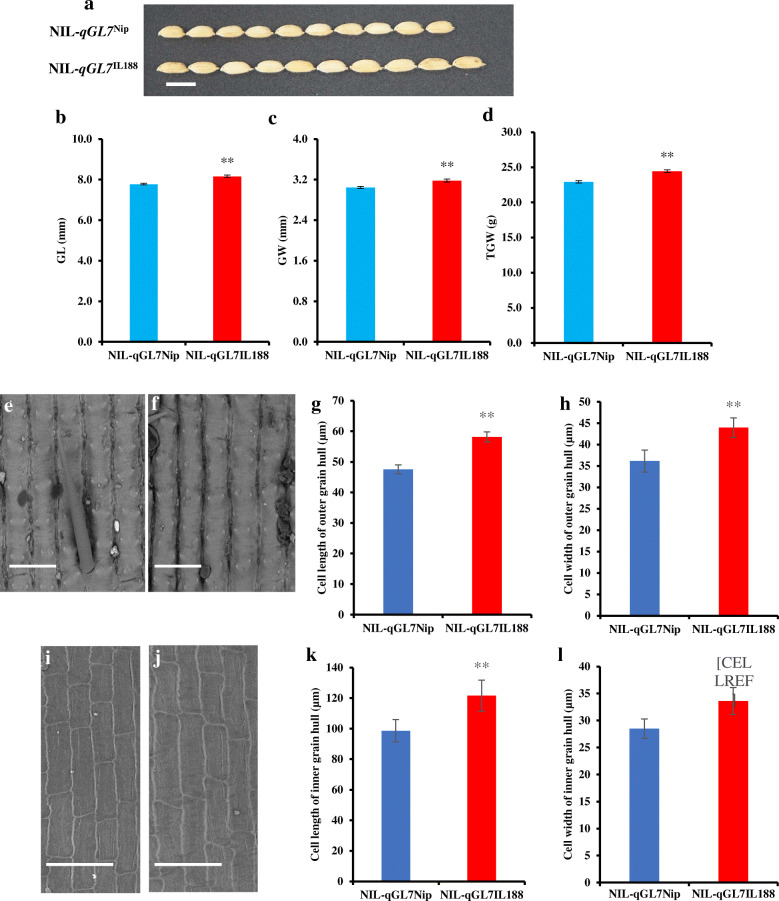
Comparison of grain size and spikelets epidermal cells between NIL-qGL7Nip and NIL-qGL7IL188. **a**, Mature grains of NIL-qGL7Nip and NIL-qGL7IL188. Scale bar, 6 mm. **b**–**d**, Grain length (GL), grain width (GW), and 1000-grain weight (TGW) for NIL-qGL7Nip and NIL-qGL7IL188. Data are given as mean ± (n = 20). ** indicate significant difference at 0.01 level. **e**–**f**, Outer epidermal cells of grain hulls of NIL-qGL7Nip and NIL-qGL7IL188. Bars = 100 μm. **g**–**h**, The average length and width of outer epidermal cells. (n = 10). **i**–**j**, Inner epidermal cells of grain hulls of NIL-qGL7Nip and NIL-qGL7IL188. Bars = 100 μm. **k**–**l**, The average length and width of inner epidermal cells (n = 10)

The homozygous plants were selected from R6 and R8. They were selfed to develop two NIL populations. The effect of *qGL7* was further validated using these two populations. Compared with NIL-*qGL7*^Nip^, the GL and GW in the NIL-*qGL7*^IL188^ were significantly larger (Fig. 5a-c), thus resulting in a larger TGW (Fig. 5d). These indicated that *qGL7* had stable effects on grain size traits.

In addition, we examined the cell length and width of epidermal cells of the outer and inner spikelet hulls of NIL-*qGL7*^Nip^ and NIL-*qGL7*^IL188^ by scanning electron microscopy. Both the length and width of epidermal cells of the outer and inner spikelet hulls were increased in NIL-*qGL7*^IL188^ compared with those in NIL-*qGL7*^Nip^ (Fig. 5e-l). Additionally, we investigated the cell number in the vertical and lateral direction of the outer spikelet hulls of the NIL-*qGL7*^Nip^ and NIL-*qGL7*^IL188^ by scanning electron microscopy. No significant difference in total cell number in the longitudinal and lateral direction of spikelet hulls was observed between NIL-*qGL7*^Nip^ and NIL-*qGL7*^IL188^ (Figure S1B-C). These results indicate that *qGL7* regulates grain size by promoting cell expansion.

## Discussion

Common wild rice is the wild ancestor of cultivated rice (Second, 1982; Oka, 1988; Wang et al. 1992). As the ancestor of cultivated rice, wild rice has been well recognized as an extremely important resource for rice improvement, since it carries many beneficial agronomic traits which have been lost in the cultivated rice through natural and human selection (Sun et al. 2001; Sakai and Itoh 2010). In the present study, an advanced backcross line, IL188, was developed from a cross between Nipponbare and *O. minuta*. The F_2_ and F_2:3_ populations derived from a cross between Nipponbare and IL188 was used to identify the QTLs controlling grain size and grain weight. A total of 12 putative QTLs for grain size and grain weight were detected in the F_2_ and F_2:3_ populations, and 9 of which were commonly detected in both populations.

A comparison of the QTL regions from this study with those seen in previous rice linkage maps (http://www.gramene.org) revealed that six regions were shared across studies. For GL and LWR, one QTL was detected in the interval RM7341–RM128 on chromosome 1. Wan et al. (2005) also detected a stable QTL for the same traits in the similar regions on chromosome 1, and Qi et al. (2017) detected a QTL for LWR closely linked with the marker RM128. One locus associated with GL and LWR (*qGL2*, and *qLWR2*) were located in the interval RM12924–RM5812 on chromosome 2. Interestingly, Yoon et al. (2006) confirmed a locus associated with TGW, GW, GT, and LWR in the same region using an advanced backcross population between *O. grandiglumis* and *O. sativa*. Two QTLs for GW and GT were respectively located in the vicinity of QTLs detected in previous studies (Yoon et al. 2006; Swamy et al. 2012; Qi et al. 2017). In our study, one QTL was detected for TGW in the interval RM6307–RM5807 on chromosome 2, and Xue et al. (2019) also detected a QTL for TGW in the nearby region.

More importantly, one major QTL, *qGL7*, was detected for GL and TGW in the interval RM500–RM429 on chromosome 7 in the F_2_ and F_2:3_ populations. The *O. minuta* introgressive line allele could increase GL and TGW. The *qGL7* could explain 15.09–16.30% and 6.65–9.97% of the phenotypic variation for GL and TGW, respectively. The *O. minuta* allele at locus *qGL7* increased GL and TGW by an average of 0.19 mm and 0.65 g, respectively. Interestingly, Rahman et al. (2007) also detected a QTL for GL and TGW in the same region using an F_2:3_ population between *O. minuta* introgression line and *O. sativa*. This result indicated that there really exist a stable QTL controlling grain size and grain weight, and the *O. minuta* allele could positively regulate grain size and grain weight. As we known, some major-effect QTLs for grain size and weight on chromosome 7 have been fine mapped and cloned in previous studies. Bai et al. (2010) detected a pleiotropic QTL for grain size and this QTL *qGL7* was narrowed down to within a 258-kb region. Shao et al. (2012) and Qiu et al. (2012) identified a major QTL *GS7*/*qSS7* on the long arm of chromosome 7 for grain size. Subsequently, This *GL7*/*GW7* (the same as *GS7*/*qSS7*) gene has been cloned by Wang et al. (2015a) and Wang et al. (2015b). They have found that copy number variations at *GL7*/*GW7* locus cause elevated expression of *GL7* and thus an increase in grain length. The grain size gene *GLW7*, encoding the plant-specific transcription factor OsSPL13, has been isolated and functionally characterized using GWAS approach (Si et al. 2016). Xu et al. (2015) identified a dominant big grain mutant BG2 that encoded a cytochrome P450, OsCYP78A13 on chromosome 7. Here, we have defined the locus *qGL7* to a 261 kb region on the long arm of chromosome 7. By comparing the physical location of *qGL7* with the reported grain size QTLs on chromosome 7, we found that *qGL7* is a novel QTL for regulating rice grain size.

Compared with the Nipponbare reference genome, seven stop code mutations and ten frameshift mutations were identified in the exonic region of the twelve genes of NIL-*qGL7*^IL188^ in the 261-kb region. Three putative genes of them, *LOC_Os07g36830*, *LOC_Os07g36900* and *LOC_Os07g36910* encoded F-box domain-containing proteins. F-box proteins are the substrate-recognition components of SCF (SKP1-Cul1-F-box) type E3 ubiquitin protein ligases (Skowyra et al. 1997; Feldman et al. 1997), which participate in the regulation of many physiological processes and play a key role in cell division, signal transduction, development and metabolism (Patton et al. 1998). In rice, *GW2* encodes a RING-type protein with E3 ubiquitin ligase activity, which is known to function in the degradation by the ubiquitin-proteasome pathway (Song et al. 2007). In addition, Chen et al. (2013) reported that overexpression of *OsFBK12* (encoding an F-box protein) could increase grain size in rice. Therefore, we suppose that the three putative genes, *LOC_Os07g36830*, *LOC_Os07g36900* and *LOC_Os07g36910* encoded F-box domain-containing proteins may be the candidate genes for *qGL7*. In future, transgenic studies will be carried out for the three F-box domain-containing genes identified in the *qGL7* locus to further elucidate the molecular mechanism of *qGL7* involving in regulation of rice grain size.

Classical quantitative genetics assumes that trait correlations are the result of either pleiotropic effects or the tight linkage of genes (Wan et al. 2005). In this study, *qGL1*/*qLWR1* and *qGL2*/*qLWR2* were mapped in the same interval on chromosome 1 and chromosome 2, respectively, and the positive alleles were all derived from *O. minuta*. As well, *qGL7* and *qTGW7* shared the same confidence interval on chromosome 7 and their effect acted in the same direction. Co-localization of these QTLs, as the result of either pleiotropic effects or close linkage, could provide an explanation for the genetic basis of high trait correlations, which ranged from 0.488 between GL and LWR to 0.614 between GL and TGW.

Transfer and utilization useful genes from wild rice into cultivated varieties are effective and aim to improve grain yield, quality, and crop genetic diversity (Brar and Khush 1997; Xie et al. 2006; Xie et al. 2008; Yun et al. 2016; Qi et al. 2017). However, efforts to improve rice grain traits of modern cultivars using *O. minuta* as donor parents are limited. In the present study, *O. minuta* alleles increase rice grain traits in the Nipponbare background at most QTLs, revealing the possibility that *O. minuta* alleles could improve grain traits. Although a number of genes/QTLs involved in the regulation of grain size have been cloned in rice, the molecular mechanisms of how grain size is regulated remain unknown. In this study, we found that *qGL7* could increase both grain length and grain weight, and the isolation of *qGL7* will be beneficial in better understanding of the regulation mechanism of grain size in rice. In addition, our continuous work will be helpful in improving rice yield and quality by molecular design breeding.

## Conclusions

An introgression line IL188 was identified, which exhibited increased grain size and weight. A total of 12 QTLs for five grain traits were detected using F_2_ and F_2:3_ populations derived from crosses between IL188 and Nipponbare. One of the QTLs, *qGL7* was delimited to a ~ 261 kb region on the long arm of chromosome 7, and three putative genes, *LOC_Os07g36830*, *LOC_Os07g36900* and *LOC_Os07g36910* encoding F-box domain-containing proteins may be the candidate genes for *qGL7*. The *qGL7* increases grain size and weight by regulating cell expansion. These results will be helpful not only for understanding the genetic basis of grain size traits, but also simultaneously improving grain size and weight through marker-assisted selection (MAS) in rice breeding programs.

## Materials and Methods

### Plant Materials

The introgression line, IL188, derived from an interspecific cross between *Oryza sativa japonica* Nipponbare and a wild species *Oryza minuta* W303 collected from the Germplasm Resource Center of IRRI, followed by three backcrosses with Nipponbare and aided by embryo rescue and subsequently self-pollinated for four generations. IL188 showed significantly longer grain length and higher grain weight than the recurrent parent Nipponbare (Fig. 1). To elucidate the genetic basis of the grain size and weight variation, an F_2_ population consisting of 166 individuals was constructed by selfing the F_1_ between the female parent IL188 and male parent Nipponbare, and the F_2:3_ population was derived from the selfed seeds of the F_2_ plants.

**Fig. 6:**
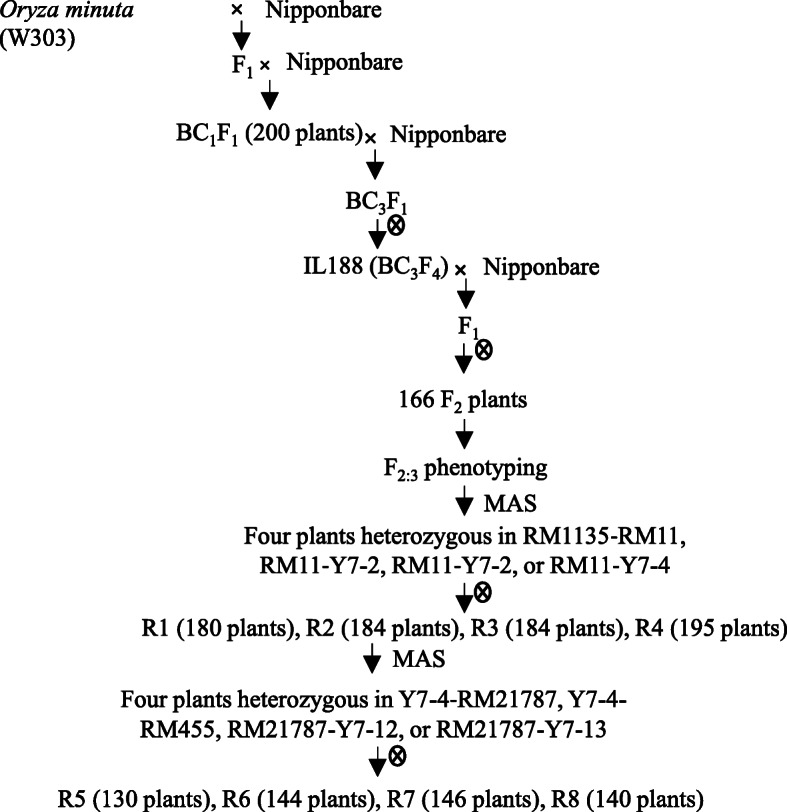
A scheme showing how plants materials were developed

Following the initial outcome of QTL analysis, four residual heterozygous plants were selected from the F_2:3_ population, carrying sequential heterozygous segments covering the interval RM500–RM429. They were selfed, and four NIL-F_2_ populations were constructed. They contained 180, 184, 184 and 195 plants and were named as R1, R2, R3 and R4, which carried overlapping heterozygous segments in the interval RM11–RM1135, RM11–Y7–2, RM11–Y7–2 and RM11–Y7–4, respectively (Fig. 6). The genomic background of the R1, R2, R3 and R4 populations was shown in Table S1. The R1, R2, R3 and R4 populations which was respectively only heterozygous in the target interval and basically homozygous in the genomic background were used for the substitution mapping of *qGL7*.

Four other plants were further selected from the R3 population carrying sequential heterozygous segments covering the interval Y7–3–Y7–4. They were selfed, and four NIL-F_2_ populations were constructed. They contained 130, 144, 146 and 140 plants and were named as R5, R6, R7 and R8, which carried overlapping heterozygous segments in the interval Y7–4–RM21787, Y7–4–RM455, RM21787–Y7–12 and RM21787–Y7–13, respectively (Fig. 6). The R5, R6, R7 and R8 populations were used for further substitution mapping of *qGL7*. Non-recombinant homozygous plants were further identified in the R6 and R8 populations and selfed. Two sets of NILs were developed, each consisting of 20 IL188 homozygous lines and 20 Nipponbare homozygous lines.

The F_2_ and F_2:3_ populations were grown at the Hangzhou Experiment Station of China Rice Research Institute (CNRRI), Zhejiang (N 30°32′, E 120°12′), China, and the Lingshui Experiment Station of CNRRI, Hainan (N 18°48′, E 110°02′), China, in the summer and winter of 2014. The NIL-F_2_ populations and two sets of NILs were planted at the Hangzhou Experiment Station of CNRRI in the summer of 2015, 2016 and 2017. The F_2_ and NIL-F_2_ populations were planted with 20 cm between plants and 30 cm between rows. The F_2:3_ families and two sets of NILs were grown in a randomized complete block design with two replications, five rows per plot, 8 plants per row, 20 cm between plants within each row and 30 cm between rows. The field management followed the standard agronomic practices.

### Grain Size Trait Evaluation

For the F_2_ and NIL-F_2_ populations, the plants were individually harvested for trait evaluation. For the F_2:3_ population and NILs-*qGL7*^Nip^ and NILs-*qGL7*^IL188^, ten plants in each line were harvested in bulk for trait evaluation. Five grain size traits were evaluated in each population. For grain length (GL), grain width (GW) and grain thickness (GT), 20 full-filled rice grains were randomly selected and individually measured using an electronic digital display vernier caliper. The averaged values of the 20 grains were used for data analysis. The grain length-width ratio (LWR) is equal to GL divided by its GW. Thousand grain weight (TGW) was evaluated by measuring the weight of 200 randomly selected full-filled grains per F_2_ plant. The phenotypic evaluations of F_2:3_ family, NIL-F_2_ population and NIL lines were the same as those for F_2_ plants described above.

### Scanning Electron Microscopy

The spikelets of NIL-*qGL7*^Nip^ and NIL-*qGL7*^IL188^ were collected at maturity stage. The samples were fixed in FAA solution (formalin: glacial acetic acid: ethanol in 1:1:18 ratio by volume) at 4 °C for 24 h, then dehydrated by a graded ethanol series, and were dried by critical-point drying method. The samples were observed under the scanning electron microscope (HITACHI, S-3000 N). The spikelet epidermal cell size was measured using image J software.

### DNA Extraction and Molecular Marker Analysis

DNA was extracted from fresh leaves samples following the CTAB method (Murray and Thompson 1980) with minor modifications. A total of 512 SSR markers with good genome coverage were selected to detect the polymorphisms between parents W303 and Nipponbare, 185 of which distributed across all 12 chromosomes showed polymorphisms between the two parents. Furthermore, 30 polymorphic SSR markers between IL188 and Nipponbare were used to genotype the F_2_ and F_2:3_ populations. Sixteen markers were used for fine mapping (Table S2).

### Linkage Map Construction and Data Analysis

A genetic linkage map was constructed using MAPMAKER/EXP version 3.0 (Lander et al. 1987). The Kosambi mapping function (Kosambi, 1944) was used to transform the recombination frequency into cM. Composite interval mapping (CIM) was carried out to scan the introgressive genomic regions for putative QTLs using Windows QTL Cartographer 2.5 (http:// statgen.ncsu.edu/qtlcart/WQTLCart.htm). The LOD threshold of 2.5 was used for declaring the presence of a putative QTL in a given genomic region. Nomenclature of QTLs was conducted as described by McCouch et al. (1997).

Phenotypic differences between IL188 and Nipponbare and between two homozygous lines in the NIL populations were compared using the student′s test. Correlation analysis of grain size traits were performed using SPSS software.

## Supplementary Information

**Additional file 1: Supplementary Table S1.** The genomic background of the R1-R4 populations. **Supplementary Table S2.** Primers used for fine mapping. **Supplementary Table S3.** The variations of NIL-*qGL7*^IL188^ in the 261-kb region compared with the Nipponbare reference genome.

**Additional file 2: Supplementary Figure S1.** Comparison of grain size and cell number in the outer spikelet hulls along the vertical and lateral direction between NIL-*qGL7*^Nip^ and NIL-*qGL7*^IL188^. Scale bar, 1 mm. A, Mature grains of NIL-*qGL7*^IL188^ (left) and NIL-*qGL7*^Nip^ (right). B-C, The cell number in the longitudinal and lateral direction of outer spikelet hulls.

## Data Availability

The datasets supporting the conclusions of this article are included within the article.

## Abbreviations

QTL: Quantitative trait locus
IL: Introgression line
RHL: Residual heterozygous line
SSR: Simple sequence repeat
GWAS: Genome wide association study
MAS: Molecular assisted selection
NIL: Near isogenic line
