# Correction to: Validation of a QTL for Grain Size and Weight Using an Introgression Line from a Cross between *Oryza sativa* and *Oryza minuta*

**DOI:** 10.1186/s12284-021-00545-1

**Published:** 2021-12-20

**Authors:** Yue Feng, Xiaoping Yuan, Yiping Wang, Yaolong Yang, Mengchen Zhang, Hanyong Yu, Qun Xu, Shan Wang, Xiaojun Niu, Xinghua Wei

**Affiliations:** Chinese National Center for Rice Improvement and State Key Laboratory of Rice Biology, China National Rice Research Institute, Hangzhou, 310006 China

## Correction to: Rice (2021) 14:43 https://doi.org/10.1186/s12284-021-00472-1

In the original publication of the article, the supplementary figure 1 was published incorrectly. The corrected supplementary figure is provided in this correction article. The original article has been corrected.

## Supplementary Information

**Additional file 1**.** Figure S1**: Comparison of grain sizeand cell number in the outer spikelet hulls along the vertical and lateraldirection between NIL-qGL7Nipand NIL-qGL7IL188. Scale bar, 1 mm. A,Mature grains of NIL-qGL7IL188(left) and NIL-qGL7Nip(right). B-C, The cellnumber in the longitudinal and lateral direction of outer spikelet hulls.
