# Supplementary material for: Validation of a QTL for Grain Size and Weight Using an Introgression Line from a Cross between Oryza sativa and Oryza minuta
Source: Rice (N Y). 2021 May 20;14:43. doi: 10.1186/s12284-021-00472-1 (PMC8137761; doi:10.1186/s12284-021-00472-1)
Supplement: Supplementary file 1 — Additional file 1: Supplementary Table S1. The genomic background of the R1-R4 populations. Supplementary Table S2. Primers used for fine mapping. Supplementary Table S3. The variations of NIL-qGL7IL188 in the 261-kb region compared with the Nipponbare reference genome. [file 12284_2021_472_MOESM1_ESM.docx]

**Table S1** The genomic background of the R1-R4 populations

| Population | Chromosome | Marker | Genotype |
| --- | --- | --- | --- |
| R1 | 1 | RM7341 | RR |
|  | 1 | RM128 | RR |
|  | 2 | RM12924 | HH |
|  | 2 | RM5812 | HH |
|  | 2 | RM6307 | HH |
|  | 2 | RM5807 | HH |
|  | 3 | RM3199 | HR |
|  | 3 | RM3684 | HR |
|  | 5 | RM289 | RR |
|  | 5 | RM18452 | RR |
|  | 6 | RM7158 | RR |
|  | 6 | RM276 | RR |
|  | 6 | RM3431 | RR |
|  | 6 | RM20071 | RR |
|  | 7 | RM20897 | HH |
|  | 7 | RM5752 | HH |
|  | 7 | RM500 | HR |
|  | 7 | RM21734 | HR |
|  | 8 | RM408 | RR |
|  | 8 | RM8243 | RR |
|  | 8 | RM3845 | RR |
|  | 8 | RM6948 | RR |
| R2 | 1 | RM7341 | HH |
|  | 1 | RM128 | HH |
|  | 2 | RM12924 | RR |
|  | 2 | RM5812 | RR |
|  | 2 | RM6307 | HH |
|  | 2 | RM5807 | HH |
|  | 3 | RM3199 | HR |
|  | 3 | RM3684 | HR |
|  | 5 | RM289 | HH |
|  | 5 | RM18452 | HH |
|  | 6 | RM7158 | RR |
|  | 6 | RM276 | RR |
|  | 6 | RM3431 | HH |
|  | 6 | RM20071 | HH |
|  | 7 | RM20897 | HH |
|  | 7 | RM5752 | HH |
|  | 7 | Y7-1 | HR |
|  | 7 | Y7-3 | HR |
|  | 8 | RM408 | HH |
|  | 8 | RM8243 | HH |
|  | 8 | RM3845 | RR |
|  | 8 | RM6948 | RR |
| R3 | 1 | RM7341 | HH |
|  | 1 | RM128 | HH |
|  | 2 | RM12924 | RR |
|  | 2 | RM5812 | RR |
|  | 2 | RM6307 | HH |
|  | 2 | RM5807 | HH |
|  | 3 | RM3199 | HR |
|  | 3 | RM3684 | HR |
|  | 5 | RM289 | HH |
|  | 5 | RM18452 | HH |
|  | 6 | RM7158 | RR |
|  | 6 | RM276 | RR |
|  | 6 | RM3431 | HH |
|  | 6 | RM20071 | HH |
|  | 7 | RM20897 | HH |
|  | 7 | RM5752 | HH |
|  | 7 | Y7-1 | HR |
|  | 7 | Y7-3 | HR |
|  | 8 | RM408 | HH |
|  | 8 | RM8243 | HH |
|  | 8 | RM3845 | RR |
|  | 8 | RM6948 | RR |
| R4 | 1 | RM7341 | HH |
|  | 1 | RM128 | HH |
|  | 2 | RM12924 | HH |
|  | 2 | RM5812 | HH |
|  | 2 | RM6307 | HH |
|  | 2 | RM5807 | HH |
|  | 3 | RM3199 | RR |
|  | 3 | RM3684 | RR |
|  | 5 | RM289 | HR |
|  | 5 | RM18452 | HR |
|  | 6 | RM7158 | RR |
|  | 6 | RM276 | RR |
|  | 6 | RM3431 | RR |
|  | 6 | RM20071 | RR |
|  | 7 | RM20897 | HR |
|  | 7 | RM5752 | HR |
|  | 7 | Y7-1 | HR |
|  | 7 | Y7-5 | HR |
|  | 8 | RM408 | HH |
|  | 8 | RM8243 | HH |
|  | 8 | RM3845 | HH |
|  | 8 | RM6948 | HH |

P_1_ = Nipponbare (HH), P_2_ = IL188 (RR), P_1_/P_2_ = Nipponbare/IL188 (HR).

**Table S2** Primers used for fine mapping

| Name | Forward primer (5´→3´) | Reverse primer (5´→3´) |
| --- | --- | --- |
| RM1135 | GTATAGCCAACCAAGCAAGATAGC | GATGCCTAGACACACATGTAAGC |
| Y7-1 | GGTGGTAACCTTGAGAGCTACG | CGAAGAGGATAGATAACCCATAGTCC |
| RM11 | ATCGGTGCTTGGCTGGATAGC | CCACCTTCTTCTCCTCCTCTTCC |
| RM21734 | AGGCTGCCGATAATGCTGTGC | GCTCCTCTCGCTGTCATGATCC |
| Y7-4 | AGTCTCCGCAACACAGAGTCAGC | TGAAGAGAAGTGCGTGATTCTTCC |
| Y7-5 | ACACGACGCAGTTTCCATTCC | CATGCTAGCTTGGATGTGTAGTGC |
| RM21787 | ACCAAGCAGAAGAGCAGCAAGC | TCGTCTTCTCCACATCAATCACC |
| RM6403 | GAACACGCCAAGAAACCAACC | TAGCACTGCGTAGCAGCAATGG |
| RM455 | CCACAAATTAATCCGGATCACACC | AGCATTGTGCAATCACGAGAAGG |
| Y7-2 | CGCACAAACAAGTAAACGACACC | CATGCTAGCTTGGATGTGTAGTGC |
| Y7-3 | ATGGGATCGTCCTCAGGGTTGG | GTGTGTGGGTGTGTGAGGTGAGG |
| RM21869 | ACTTGGGCCACGCACAGAAACC | GTGGTAGTGGGAGAGGAGAGGAGAGG |
| RM6098 | GCCGCCATGAGCAACAGAGC | TGGCTGCGAGGAAGAAGAACTAGC |
| Y7-12 | ACGCCTTCTCGCACGAGTT | AGCGAGAGGAACAACACGAC |
| Y7-13 | GAGAACGGCTTCTGGATGTG | CGCTGTTCATGGTCGTCTAC |
| Y7-38 | AATTCACCGCTCTCCAGTCTCC | TCTCCGTATAGAGCGAGGTAGGG |

**Table S3** The variations of NIL-*qGL7*^IL188^ in the 261-kb region compared with the Nipponbare reference genome

| Mutation_type | POS_strt | POS_end | Ref | Alt | GeneID | Chr7 | start | end | Description |
| --- | --- | --- | --- | --- | --- | --- | --- | --- | --- |
| stopgain | 21998892 | 21998892 | G | A | LOC_Os07g36720 | Chr7 | 21997650 | 22002266 | retrotransposon protein, putative, unclassified, expressed |
| stoploss | 22029087 | 22029087 | A | G | LOC_Os07g36770 | Chr7 | 22029085 | 22034860 | retrotransposon protein, putative, Ty3-gypsy subclass, expressed |
| stoploss | 22071294 | 22071294 | T | C | LOC_Os07g36830 | Chr7 | 22066415 | 22071296 | OsFBX250 - F-box domain containing protein, expressed |
| stoploss | 22076761 | 22076761 | T | C | LOC_Os07g36850 | Chr7 | 22075841 | 22076763 | transposon protein, putative, Pong sub-class, expressed |
| stopgain | 22097827 | 22097827 | G | A | LOC_Os07g36900 | Chr7 | 22095130 | 22099277 | OsFBL39 - F-box domain and LRR containing protein, expressed |
| stopgain | 22102720 | 22102720 | G | A | LOC_Os07g36910 | Chr7 | 22101917 | 22105152 | OsFBX254 - F-box domain containing protein, expressed |
| stopgain | 22102895 | 22102895 | C | T | LOC_Os07g36910 | Chr7 | 22101917 | 22105152 | OsFBX254 - F-box domain containing protein, expressed |
| frameshift deletion | 21870069 | 21870069 | C | - | LOC_Os07g36580 | Chr7 | 21870046 | 21872441 | expressed protein |
| frameshift deletion | 21942103 | 21942104 | GC | - | LOC_Os07g36660 | Chr7 | 21939836 | 21943033 | retrotransposon protein, putative, unclassified, expressed |
| frameshift insertion | 21944285 | 21944285 | - | A | LOC_Os07g36670 | Chr7 | 21943658 | 21949282 | retrotransposon protein, putative, unclassified, expressed |
| frameshift deletion | 21948986 | 21948986 | G | - | LOC_Os07g36670 | Chr7 | 21943658 | 21949282 | retrotransposon protein, putative, unclassified, expressed |
| frameshift deletion | 21955733 | 21955733 | C | - | LOC_Os07g36680 | Chr7 | 21952841 | 21956478 | expressed protein |
| frameshift insertion | 21998471 | 21998471 | - | G | LOC_Os07g36720 | Chr7 | 21997650 | 22002266 | retrotransposon protein, putative, unclassified, expressed |
| frameshift deletion | 22066562 | 22066565 | ACCG | - | LOC_Os07g36830 | Chr7 | 22066415 | 22071296 | OsFBX250 - F-box domain containing protein, expressed |
| frameshift deletion | 22066849 | 22066849 | C | - | LOC_Os07g36830 | Chr7 | 22066415 | 22071296 | OsFBX250 - F-box domain containing protein, expressed |
| frameshift insertion | 22088763 | 22088763 | - | G | LOC_Os07g36880 | Chr7 | 22088000 | 22090410 | expressed protein |
| frameshift insertion | 22126254 | 22126254 | - | G | LOC_Os07g36950 | Chr7 | 22125139 | 22127924 | expressed protein |
