# Supplementary figures and images for: Validation of a QTL for Grain Size and Weight Using an Introgression Line from a Cross between Oryza sativa and Oryza minuta
Source: Rice (N Y). 2021 May 20;14:43. doi: 10.1186/s12284-021-00472-1 (PMC8137761; doi:10.1186/s12284-021-00472-1)

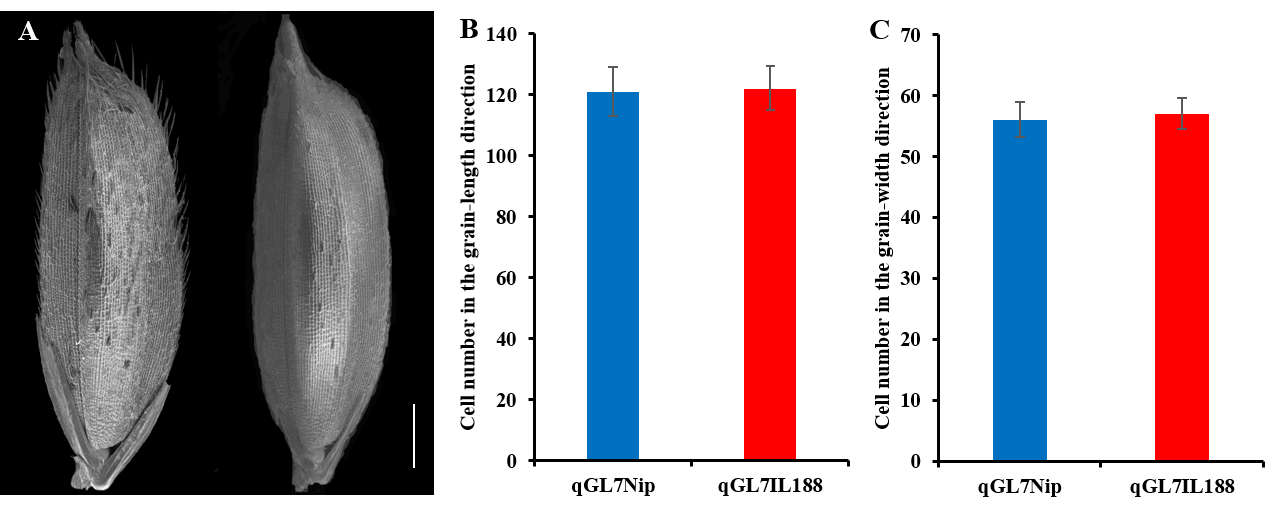

Supplement: Supplementary file 2 — Additional file 2: Supplementary Figure S1. Comparison of grain size and cell number in the outer spikelet hulls along the vertical and lateral direction between NIL-qGL7Nip and NIL-qGL7IL188. Scale bar, 1 mm. A, Mature grains of NIL-qGL7IL188 (left) and NIL-qGL7Nip (right). B-C, The cell number in the longitudinal and lateral direction of outer spikelet hulls. [file 12284_2021_472_MOESM2_ESM.tif]
